# Identifying hub genes and common biological pathways between COVID-19 and benign prostatic hyperplasia by machine learning algorithms

**DOI:** 10.3389/fimmu.2023.1172724

**Published:** 2023-06-23

**Authors:** Hang Zhou, Mingming Xu, Ping Hu, Yuezheng Li, Congzhe Ren, Muwei Li, Yang Pan, Shangren Wang, Xiaoqiang Liu

**Affiliations:** ^1^ Department of Urology, Tianjin Medical University General Hospital, Tianjin, China; ^2^ Department of Orthopedics, Tianjin Medical University General Hospital, Tianjin, China

**Keywords:** COVID-19, benign prostatic hyperplasia, functional enrichment, hub genes, machine learning algorithms

## Abstract

**Background:**

COVID-19, a serious respiratory disease that has the potential to affect numerous organs, is a serious threat to the health of people around the world. The objective of this article is to investigate the potential biological targets and mechanisms by which SARS-CoV-2 affects benign prostatic hyperplasia (BPH) and related symptoms.

**Methods:**

We downloaded the COVID-19 datasets (GSE157103 and GSE166253) and the BPH datasets (GSE7307 and GSE132714) from the Gene Expression Omnibus (GEO) database. In GSE157103 and GSE7307, differentially expressed genes (DEGs) were found using the “Limma” package, and the intersection was utilized to obtain common DEGs. Further analyses followed, including those using Protein-Protein Interaction (PPI), Gene Ontology (GO) function enrichment analysis, and the Kyoto Encyclopedia of Genes and Genomes (KEGG). Potential hub genes were screened using three machine learning methods, and they were later verified using GSE132714 and GSE166253. The CIBERSORT analysis and the identification of transcription factors, miRNAs, and drugs as candidates were among the subsequent analyses.

**Results:**

We identified 97 common DEGs from GSE157103 and GSE7307. According to the GO and KEGG analyses, the primary gene enrichment pathways were immune-related pathways. Machine learning methods were used to identify five hub genes (BIRC5, DNAJC4, DTL, LILRB2, and NDC80). They had good diagnostic properties in the training sets and were validated in the validation sets. According to CIBERSORT analysis, hub genes were closely related to CD4 memory activated of T cells, T cells regulatory and NK cells activated. The top 10 drug candidates (lucanthone, phytoestrogens, etoposide, dasatinib, piroxicam, pyrvinium, rapamycin, niclosamide, genistein, and testosterone) will also be evaluated by the *P* value, which is expected to be helpful for the treatment of COVID-19-infected patients with BPH.

**Conclusion:**

Our findings reveal common signaling pathways, possible biological targets, and promising small molecule drugs for BPH and COVID-19. This is crucial to understand the potential common pathogenic and susceptibility pathways between them.

## 1 Introduction

An infectious disease, COVID-19, caused by the SARS Coronavirus 2 (SARS-CoV-2), poses a major danger to worldwide public health (1, 2). The most common symptom of COVID-19 is pneumonia, with severe cases frequently developing life-threatening acute respiratory distress syndrome and respiratory failure, along with fever, sore throat, difficulty breathing and coughing (1, 3). Symptoms can worsen and lead to respiratory failure, which is potentially fatal and affects the heart, liver, neurological system, and kidneys (4–7). The percentage of patients with COVID-19 who develop gastrointestinal symptoms such as nausea, diarrhea, bloating, and bleeding ranges from 3% to 40.7% (8). With the advent of vaccines (9–11) and antiviral drugs (12, 13), the spread and fatality rate of COVID-19 have decreased, but with the advent of new variations such as Delta and Omicron, it continues to pose dangers and difficulties to global health (14, 15).

In older men, benign prostatic hyperplasia (BPH), which causes benign prostate enlargement due to uncontrolled expansion of epithelial and fibromuscular tissue in the migratory zone of the urinary tract and urethral region, is a frequent disorder (16, 17). Lower urinary tract symptoms (LUTS) are common in older men and include frequent urination, inadequate urine flow, delayed urine flow, and nocturia, all of which have a negative influence on quality of life (17). According to a meta-analysis, the lifetime prevalence of BPH is 26.2% (18). Previous research suggests that elderly men may have a higher risk of developing BPH. Older men appear to have more severe cases of COVID-19 and are more likely to infect SARS-CoV-2 (19). Many patients with COVID-19 have experienced serious urinary problems (20, 21). LUTS may be one of the symptoms of COVID-19, and SARS-CoV-2 virus infection may aggravate symptoms in elderly patients with BPH (22, 23). There has not yet been any pertinent research on the possible mode of action between COVID-19 and the symptoms of BPH. To provide novel assistance for the diagnosis and treatment of disorders that have both COVID-19 and BPH, it is necessary to investigate potential hub genes and molecular pathways.

By comparing gene expression across disease groups and healthy tissues, it is now possible to explore the potential pathophysiology of many diseases, thanks to the rapid advance of gene sequencing technologies and bioinformatics analytic techniques. In addition to logistic regression with the least absolute shrinkage and selection operator (LASSO) (24), machine learning techniques such as support vector machine recursive feature elimination (SVM-RFE) (25) and random forest (RF) algorithms (26) are frequently used to accurately identify diagnostic indicators and prediction models. Several studies have been conducted to date to find hub genes and possible biomarkers using different machine learning methods (27, 28).

We attempted to discover common differentially expressed genes (DEGs) in this work by integrating the analysis of the COVID-19 dataset (GSE157103) with the BPH dataset (GSE7307). To identify probable pathways, we used the functional enrichment analysis of gene ontology (GO) and the Kyoto Encyclopedia of Genes and Genomes (KEGG). We also constructed protein-protein interactions (PPI) networks. After that, we used three different machine learning algorithms to find relevant biomarkers and examine their diagnostic value in patients with COVID-19 and BPH. For validation, we additionally used the data sets GSE166253 and GSE132714. The CIBERSORT tool was also applied to calculate the proportion of COVID-19 immune cell infiltration. Finally, we predicted transcription factors (TFs), miRNAs, and small molecule drugs.

## 2 Materials and methods

### 2.1 Data acquisition

Four datasets, including two COVID-19 datasets and two BPH datasets, were retrieved from the Gene Expression Omnibus (GEO) database. The training set used GSE157103, which contains 100 samples from COVID-19 patients and 26 samples from controls, as it has a sample size that is significantly larger than GSE166253. The other dataset, GSE166253, which included 10 samples each of COVID-19 patients and healthy individuals, served as a validation set. Because the control sample of GSE132714 is too small, we did not consider it the training set. A BPH dataset, GSE7307, which consists of 7 BPH patients and 12 healthy controls, was used as the training dataset, and another BPH dataset, GSE132714, which consists of 12 BPH patients and 4 healthy controls, was used as a verification set.

### 2.2 Identification of common DEGs

We applied false discovery rate (FDR) to adjust the *P*-value. With adj. *P* < 0.05 and |log_2_FC| > 0.263 for GSE157103 and adj. *P* < 0.05 and |log_2_FC| > 1 for GSE7307, the DEGs were found using the “Limma” R package (29). Heatmaps and volcano plots were created using the “pheatmap” and “ggplot2” tools. Common DEGs of COVID-19 and BPH were obtained through the Venn diagram.

### 2.3 Functional and pathway enrichment analysis

We analyzed the KEGG and GO enrichment items of common DEGs using the “ClusterProfiler” package (30). GO analysis included three subcategories: molecular function (MF), biological process (BP), and cellular component (CC). Additionally, to select relevant pathways, we employed an adjusted statistical threshold criterion of *P* < 0.05.

### 2.4 Construction of PPI networks

We created PPI networks to demonstrate protein interactions, which are critical to understanding the physiology of cellular physiology in health and disease at the protein level. We used STRING (https://www.string-db.org/) (version 11.5) to create PPI networks that hide unconnected nodes (31). Subsequently, Cytoscape (version 3.9.1) was utilized for visual display.

### 2.5 Using three machine learning algorithms to identify hub genes

The three most common machine learning algorithms used for disease identification and prediction are the RF algorithm, LASSO regression, and SVM-RFE technique. They can help us find the hub genes. The dimensional significance values were determined using the diminishing accuracy approach (Gini coefficient method) using a random forest model (32). The best random forest tree count was 500, and disease-specific genes were identified in the top 15 for significance value. After that, LASSO regression analysis using putative pivotal genes was conducted using the “glmnet” R package to find significant combinations of predicted genes that are consistently connected with COVID-19 (33). In this study, we used the cv.glmnet function here to select the optimal λ value by ten-fold cross-validation. Based on the output, we obtain two λ values: lambda.min=0.01010184 and lambda.1se=0.0212634. We used the value of 0.01010184 to get the coefficients of the final LASSO model, because it makes the cross-validation error minimal. The “e1071” package was applied to perform the SVM-RFE algorithm to find important genes (34). A supervised learning model, called the SVM-RFE, accurately categorizes data points by maximizing the separation between two hyperplanes. The final step was intersecting the possible genes identified by the RF, SVM-RFE, and LASSO algorithms. The overlapping genes were then used as hub genes and displayed by the Venn diagram.

### 2.6 Evaluation of expression levels and diagnostic value of hub genes

In the COVID-19 training set (GSE157103), analyses of hub genes expression were conducted. The ROC curves were then plotted using GraphPad Prism 9, and to evaluate the prediction effectiveness, the area under the curve (AUC) was best evaluated. The results were then validated using the validation set GSE166253. These genes were strongly predictive for the diagnosis of COVID-19, according to AUC > 0.6 and *P* < 0.05. ROC curves were developed using the BPH training set (GSE7307) and the validation set (GSE132714) to examine the diagnostic efficacy of hub genes for BPH.

### 2.7 Immune cell infiltration analysis

To explore the extent of different immune cell infiltration, the CIBERSORT algorithm was utilized to categorize and count the 22 categories of immune cells in the COVID-19 and control groups (35). Ultimately, the link between genes and immune infiltration was discovered using Spearman’s correlation analysis (36).

### 2.8 Prediction of transcription factors (RFs), MiRNAs and small-molecule drugs

We searched the ChEA database for transcription factor (TF)-gene interactions using the NetworkAnalyst platform (www.networkanalyst.ca) (37). Similarly, we used this platform to search the Tarbase database (version 8.0) for miRNA-gene interactions (38). The results were then visualized using Cytoscape. DSigDB is a gene set database that is linked to medications/compounds. The Enrichr platform (https://maayanlab.cloud/Enrichr/) was applied to access the DSigDB database (39), and small molecule drugs were predicted by entering the names of hub genes.

### 2.9 Identification of disease association

The DisGeNet database is one of the most extensive databases of human disease-related genes and variations (40). To discover associated diseases and chronic health conditions, we used the DisGeNET database in the NetworkAnalyst platform.

## 3 Results

### 3.1 Identification of common DEGs

**Figure 1:**
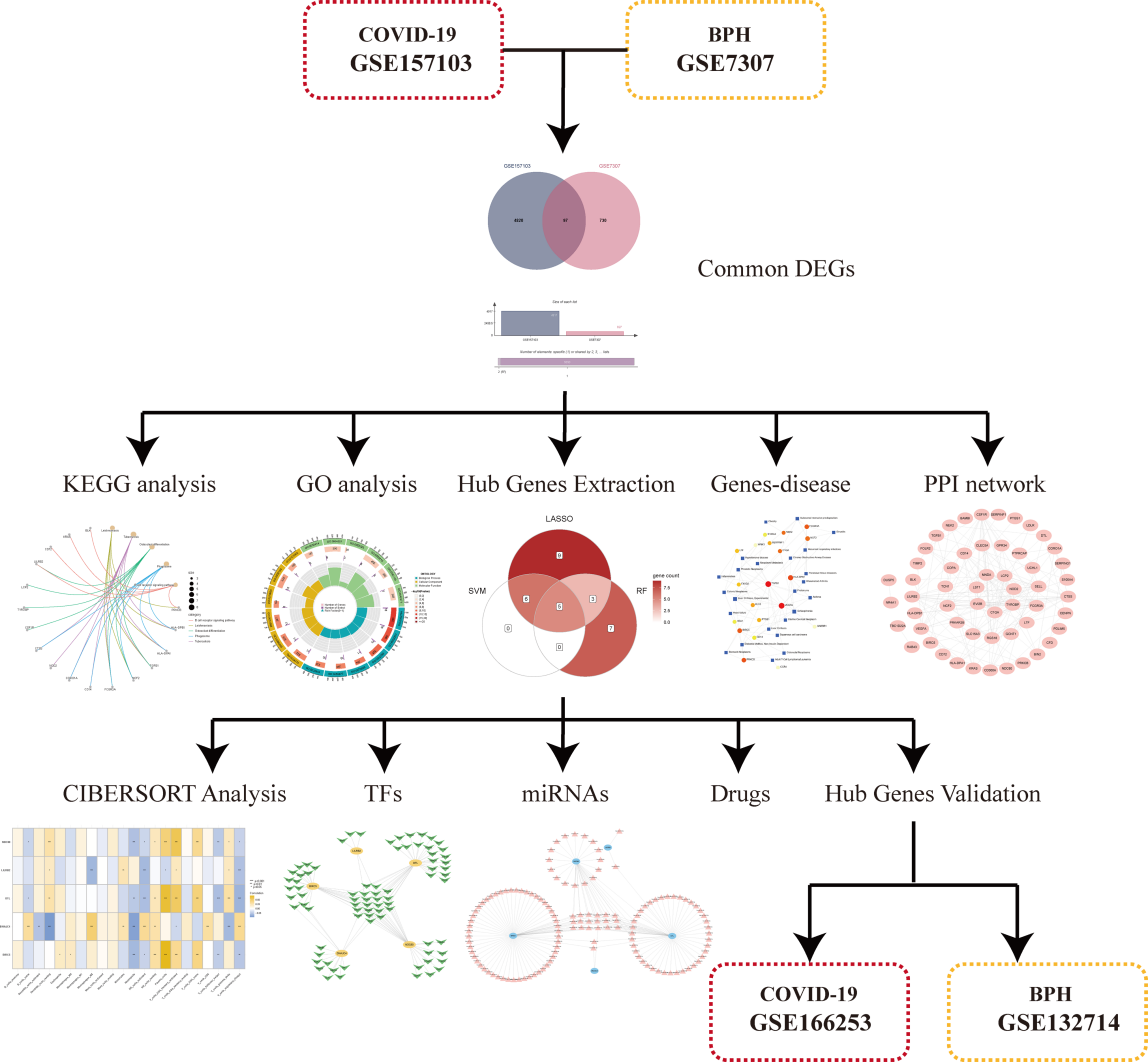
The general work flow chart of this study.

**Figure 2:**
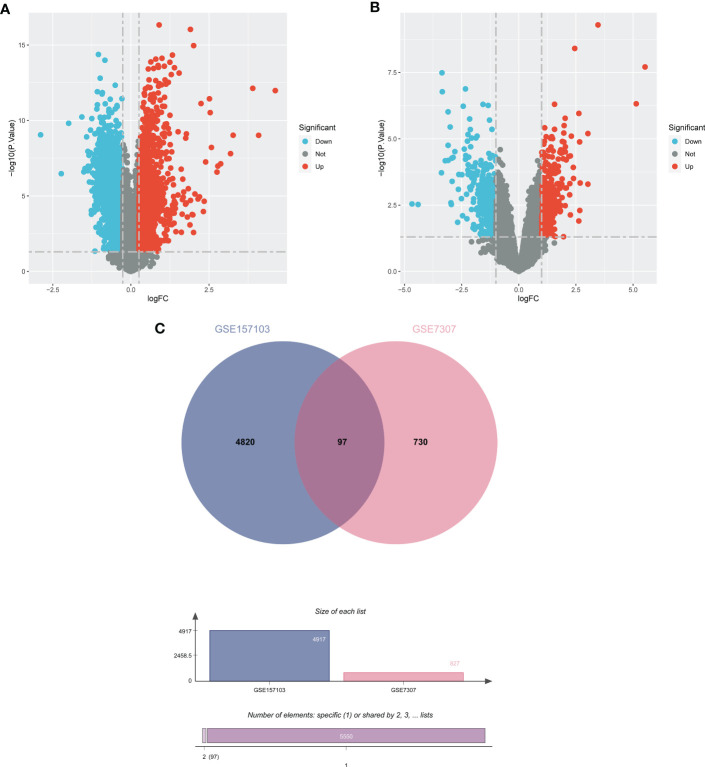
The volcano plots show DEGs of **(A)** COVID-19 (GSE157103) and **(B)** BPH (GSE7307) and **(C)** the Venn diagram of common DEGs.

In order to demonstrate the entire analysis process, we created a flow chart (**Figure 1**). In the GSE157103, we identified 4917 DEGs, including 2924 up-regulated genes and 1993 down-regulated genes (**Figure 2A**). In GSE7307, we identified 827 DEGs, including 419 up-regulated genes and 408 down-regulated genes (**Figure 2B**). Heatmaps were interpreted for DEGs in COVID-19 and BPH, respectively (**Figure S1**). According to the Venn diagram, 97 common DEGs were discovered in both the GSE157103 and the GSE7307 (**Figure 2C**).

### 3.2 Functional and pathway enrichment analysis

**Figure 3:**
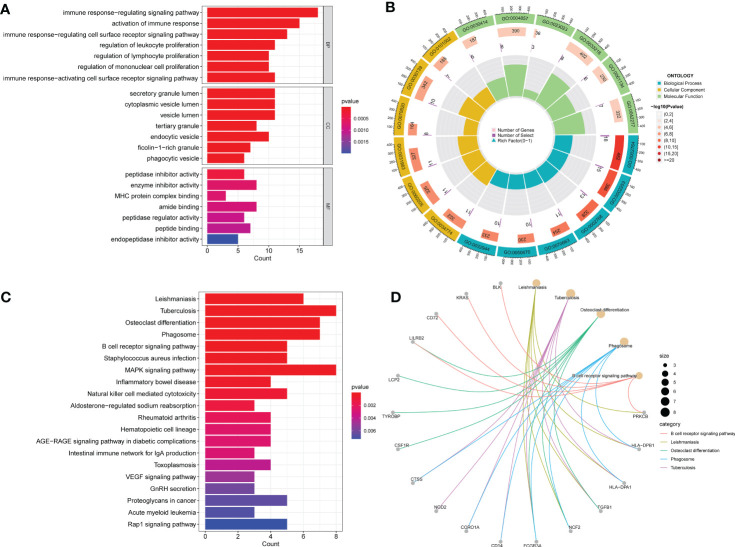
GO and KEGG functional enrichment analysis of the common DEGs between COVID-19 and BPH. **(A)** The bar plot of GO enrichment analysis. **(B)** The circle diagram of the GO enrichment analysis. **(C)** The bar plot of KEGG enrichment analysis. **(D)** The loop graph of the KEGG enrichment analysis.

GO analysis indicated significantly enriched pathways, including BP, CC, and MF (**Figures 3A, B**). Significant pathways in the BP category were the immune response-regulating signaling pathway, activation of the immune response, and the immune response regulating cell surface receptor signaling pathway. The main terms in the CC category are the secretory granule lumen, the cytoplasmic vesicle lumen, and the vesicle lumen. Furthermore, in the MF category, the main terms of statistical significance were enzyme activity inhibitor, amide binding, and peptide binding. KEGG analysis revealed the B cell receptor signaling pathway, inflammatory bowel disease, the MAPK signaling pathway, and cytotoxicity mediated by natural killer cells (**Figures 3C, D**). Our findings showed that these common DEGs are linked to inflammation and immune cells.

### 3.3 Construction of PPI network

**Figure 4:**
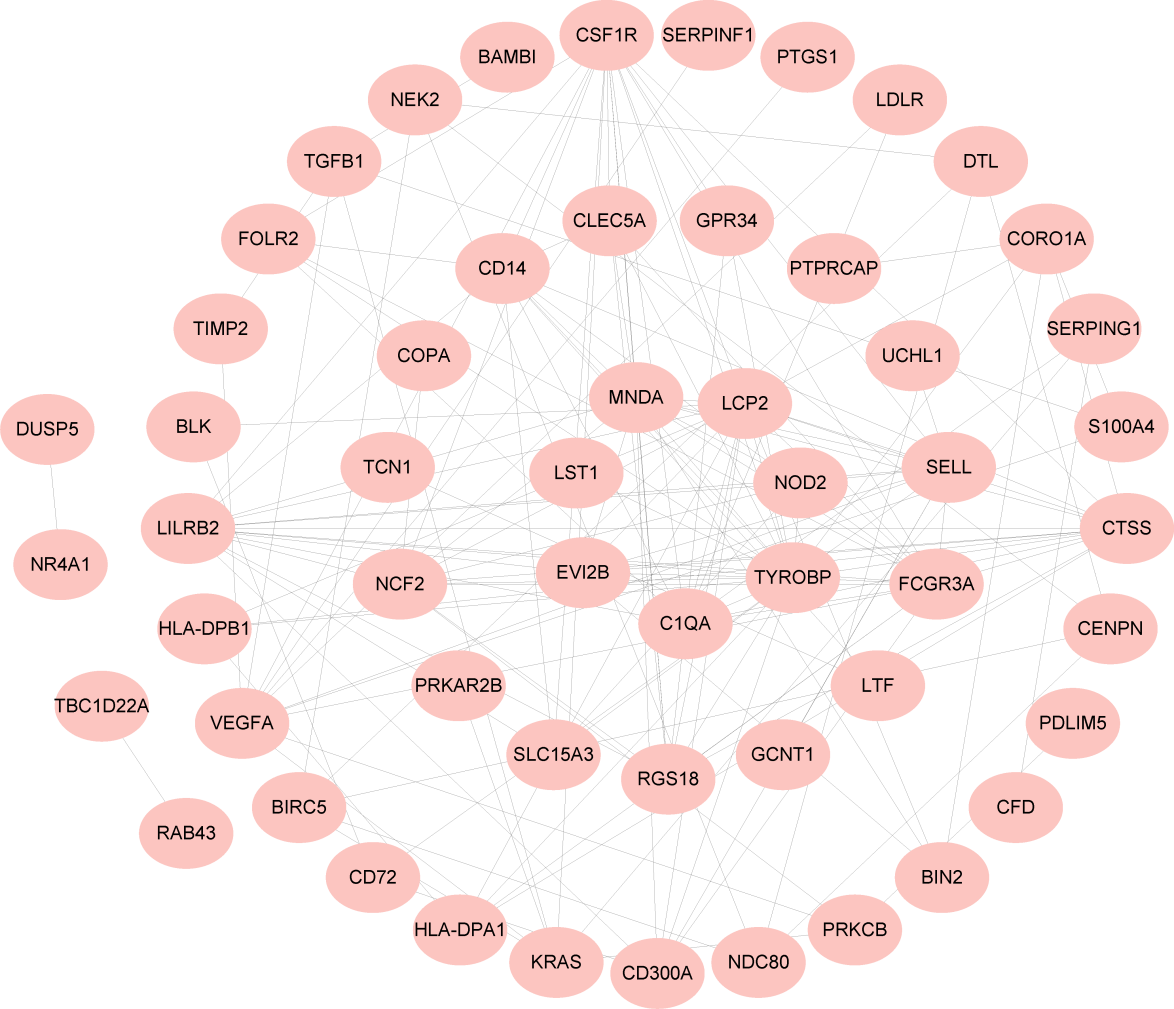
COVID-19 and BPH common DEGs in the PPI network.

A PPI network of 97 common DEGs was generated using the STRING online site to find protein interactions and visualized using Cytoscape software (**Figure 4**).

### 3.4 Using three machine learning algorithms to identify hub genes

**Figure 5:**
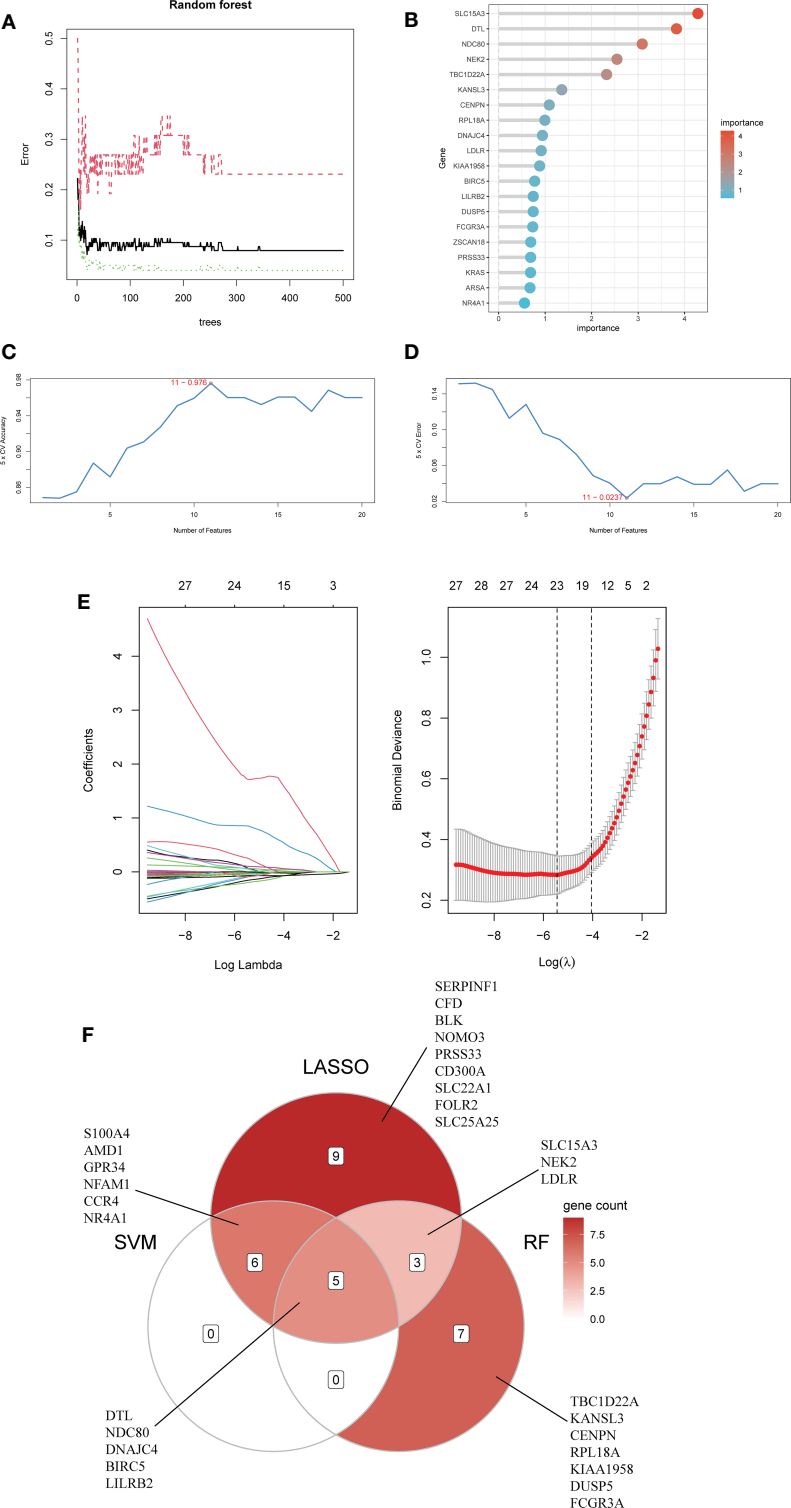
Using Random Forest (RF) to screen characteristic genes from common DEGs: **(A)** The random forest trees; **(B)** The importance rankings of features. **(C, D)** The SVM model with the highest accuracy and lowest error rate was established on 11 characteristic genes. **(E)** The establishment of LASSO model. **(F)** The Venn diagram of hub genes identified by three machine algorithms.

First, we used the RF algorithm to narrow the range to 97 DEGs. Recursive random forest classification was performed for all possible values of 1-97 variables, and the average error rate of the model was assessed for all chosen variables, as shown in **Figure 5A**. Secondly, we examined the link between model error and the number of decision trees. Finally, we chose the top 15 genes in terms of importance (SLC15A3, DTL, NDC80, NEK2, TBC1D22A, KANSL3, CENPN, RPL18A, DNAJC4, LDLR, KIAA1958, BIRC5, LILRB2, DUSP5, FCGR3A) as the likely genes for further investigation (**Figure 5B**).

The SVM model based on 11 signature genes got the best accuracy (0.976) and the lowest error rate (0.024) (**Figures 5C, D**). Therefore, 11 genes, including S100A4, DTL, LILRB2, AMD1, BIRC5, NDC80, GPR34, DNAJC4, NFAM1, CCR4, and NR4A1, were potential genes. Using LASSO regression analysis, 23 common specific genes were finally found, including GPR34, DTL, SERPINF1, NEK2, NR4A1, S100A4, CFD, LDLR, NDC80, BLK, NOMO3, SLC15A3, NFAM1, AMD1, PRSS33, BIRC5, LILRB2, CD300A, CCR4, SLC22A1, FOLR2, SLC25A25, and DNAJC4 (**Figure 5E**). Finally, according to the results of the intersection of the analysis of three machine learning methods, five hub genes (BIRC5, DNAJC4, DTL, LILRB2, and NDC80) were identified using a Venn diagram (**Figure 5F**).

### 3.5 Evaluation of expression levels and diagnostic value of hub genes

**Figure 6:**
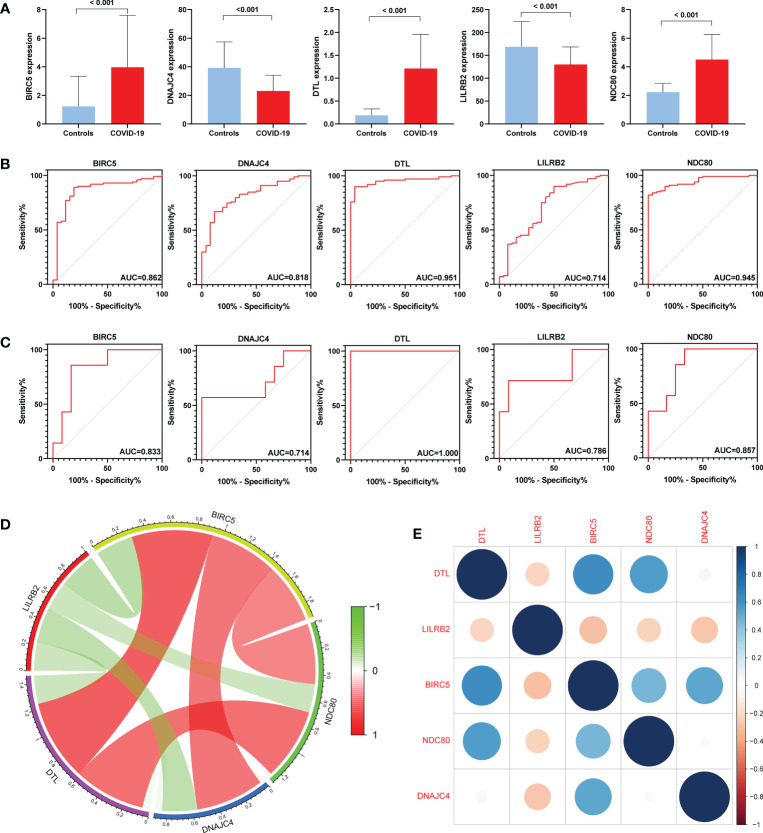
Expression levels and diagnostic significance of hub genes. **(A)** Expression levels in the COVID-19 set (GSE157103). **(B)** ROC curves in GSE157103. **(C)** ROC curves in GSE7307. **(D)** Constructing co-expression network of hub genes in COVID-19. **(E)** Heatmap of the hub genes in COVID-19.

First, we compared the expression levels of five genes in COVID-19 and controls, finding that the expression of BIRC5, DTL, and NDC80 increased in COVID-19 while DNAJC4 and LILRB2 decreased (**Figure 6A**). Subsequently, ROC analysis was performed on the BPH and COVID-19 training sets. The area under the curve (AUC) value for all five genes in GSE7307 was greater than 0.714, as was the AUC value for all five genes in GSE157103 (**Figures 6B, C**). ROC analysis of the COVID-19 validation set (GSE166253) showed that the AUC area of five genes was greater than 0.670 (**Figure S2**). ROC analysis of the BPH validation set (GSE132714) showed that only four hub genes had AUC areas greater than 0.6, while BIRC5 had an AUC area of 0.542 (**Figure S3**). The results of the ROC analysis concluded that these hub genes have excellent diagnostic properties for COVID-19 and BPH. In addition, co-expression networks of five hub genes were constructed through gene co-expression network analysis, and gene correlation heatmaps were also built (**Figures 6D, E**).

### 3.6 Immune cell infiltration analysis

**Figure 7:**
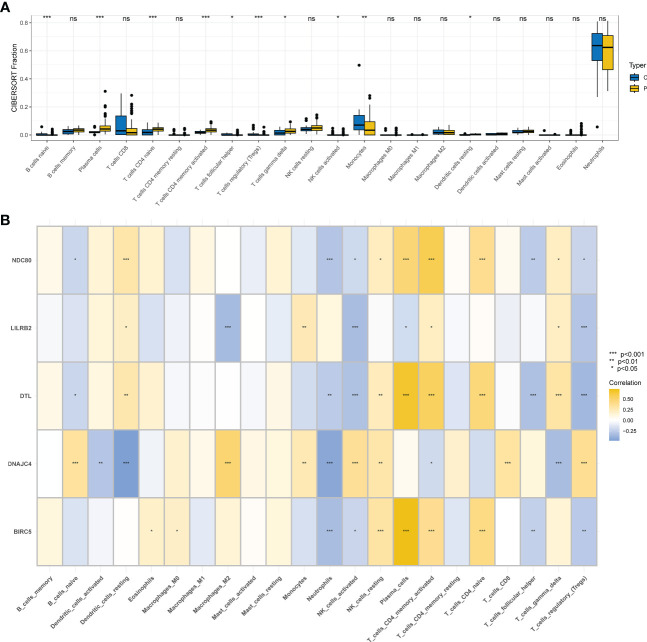
Immunity infiltration analysis based on the CIBERSORT algorithm. **(A)** Box plot of 22 types of immunity infiltrating cells in the COVID-19 and controls. **(B)** The correlation between five hub genes and immune cells.

Ten different types of immune cells were significantly different between COVID-19 and controls according to the CIBERSORT analysis of GSE157103. Five of them are associated with T cells: T cells gamma delta, T cells CD4 naive, T cells CD4 memory activated, T cells follicular helper, and T cells regulatory (Tregs) (**Figure 7A**). First, BIRC5, DTL, LILRB2, and NDC80 showed positive correlations with CD4 memory activated T cells by the correlation analysis of 5 hub genes and immune cells, but DNAJC4 showed negative correlations with CD4 memory activated T cells. Nevertheless, we found that BIRC5, DTL, LILRB2, and NDC80 had a negative correlation with activated Tregs and NK cells, but DNAJC4 had a positive correlation with them (**Figure 7B**).

### 3.7 Prediction of key TFs, MiRNAs, and small-molecule drugs

TFs and miRNAs are two different categories of gene expression regulators. A total of 79 TFs and 5 hub genes were included in the regulatory network of TFs and hub genes that we first examined. The top 10 TFs were ranked according to the betweenes, and the top 10 TFs were SPI1, POU5F1, MYBL2, PDX1, CREB1, CREM, MYC, KDM5B, E2F4, and MYCN (**Figure S4**). 130 miRNAs and 5 genes in total were engaged in the study of the gene-miRNA regulation network (**Figure S5**). The above regulatory network suggests a strong correlation between hub genes, TFs and miRNAs.

The DSigDB database was applied to predict small molecule drugs for five hub genes, and the top ten drugs by p-value were lucanthone, phytoestrogens, etoposide, dasatinib, piroxicam, pyrvinium, rapamycin, niclosamide, genistein, and testosterone (**Table 1**). These identified small molecule compounds may be potential therapeutics for COVID-19 and BPH.

**Table 1:** Drug candidates (top ten) identified by gene-drug interaction analysis.

Name	P-value	Combined Score
lucanthone	1.17E-05	1604.544241
phytoestrogens	5.61E-05	2829.762918
etoposide	5.61E-05	2829.762918
dasatinib	1.51E-04	515.4745901
piroxicam	1.97E-04	455.7848279
pyrvinium	2.07E-04	1250.437687
rapamycin	0.001482212	350.0839021
niclosamide	0.00172901	315.690249
genistein	0.002117354	141.1556855
testosterone	0.002198304	138.3730945

### 3.8 Identification of disease association

**Figure 8:**
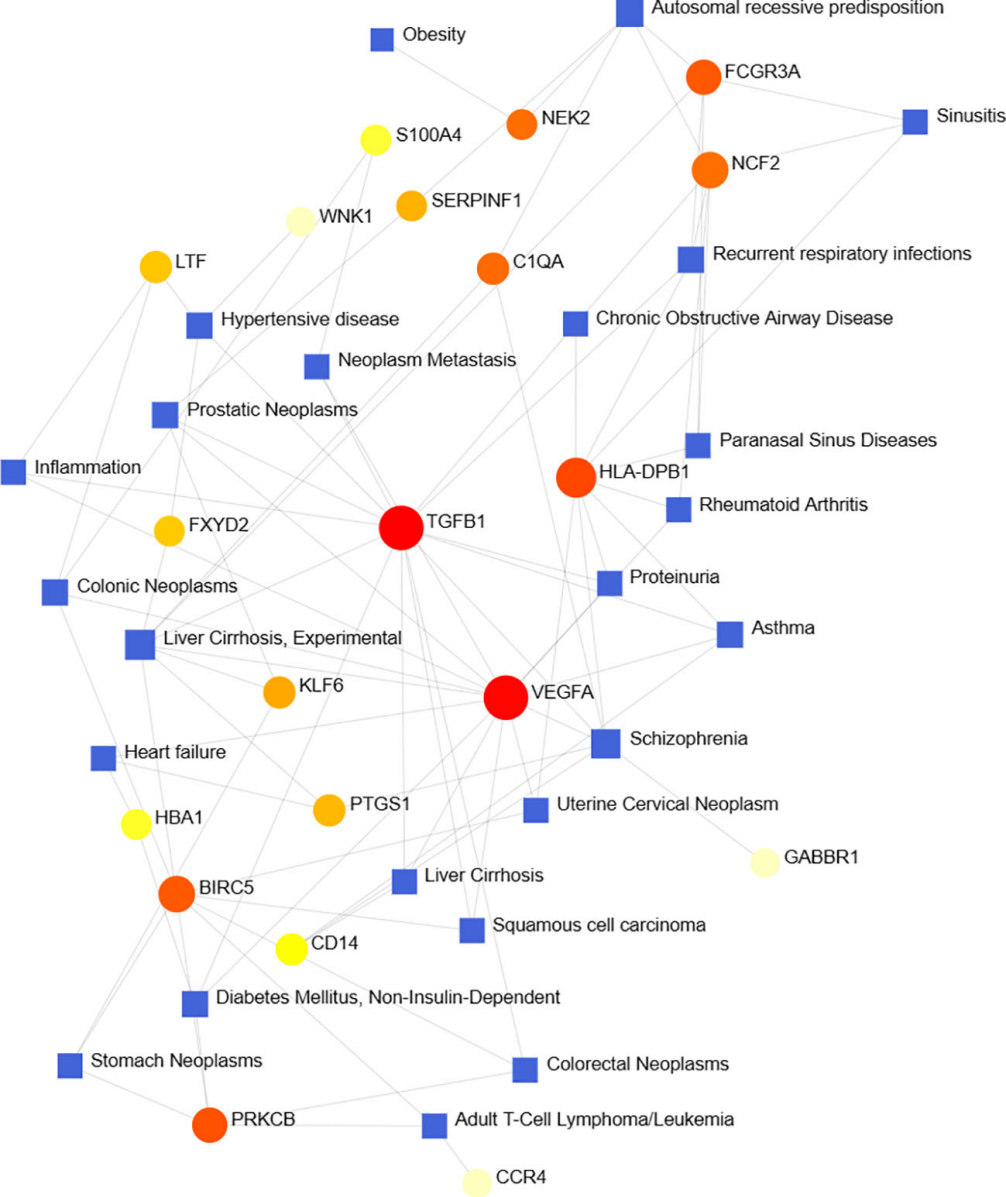
Gene-disease association network shows diseases associated with DEGs.

According to previous studies, various diseases are interrelated, and there are common genes (41). We filtered the top ten closely related diseases by importing 97 DEGs into the DisGeNet database and classified them by degree, including liver cirrhosis, schizophrenia, autosomal recessive predisposition, prostatic neoplasms, hypertensive disease, recurrent respiratory infections, diabetes mellitus, asthma, colonic neoplasms and adult T-cell lymphoma/leukemia (**Figure 8**).

## 4 Discussion

Many investigations have been conducted since the COVID-19 pandemic to support the theory that many diseases may be correlated with COVID-19 (42–46). As indicated, many male patients were found to have LUTS during COVID-19 clinical therapy of COVID-19, which may be related to BPH. Nevertheless, as of now, we don’t know enough about COVID-19 and BPH. This study sought to identify crucial genes and biological mechanisms that connect COVID-19 to BPH. Using the COVID-19 dataset (GSE157103) and the BPH dataset (GSE7307), we were able to identify 97 common DEGs. We carried out a functional enrichment analysis using KEGG and GO analyses. Five genes (BIRC5, DNAJC4, DTL, LILRB2, and NDC80) were then identified as possible hub genes by three machine learning methods (LASSSO regression, SVM-RFE, and RF).

Using DEGs enrichment analysis, we can better understand the precise mechanisms of action and the regulatory function of genes in the human body. According to KEGG data, these genes seem to be abundant in pathways related to inflammation and infection, including the B-cell receptor signaling pathway, inflammatory bowel disease, and cytotoxicity mediated by natural killer cells. The role of B cells in immunity to SARS-CoV-2 infection and vaccination has been demonstrated in several studies, and the type of SARS-CoV-2 exposure has distinct effects on the formation of B cell receptors (47–50). Russell et al. thought that the spleens of COVID-19 patients had higher levels of some components of the B cell signaling pathway (51). Following SARS-CoV-2 infection, NK cell-mediated cytotoxicity can be reduced as a result of mechanisms that result in a marked reduction in CD16/56+ NK cells and may be related to the severity of the illness or by up-regulation of an inhibitory receptor that regulates NK cell-mediated cytotoxicity (52). Creatinine may boost NK cell-mediated cytotoxic actions to treat individuals with mild to moderate COVID-19, according to recent randomized controlled research (53). Wang et al. confirmed that COVID-19 can cause testicular cell senescence via the MAPK signaling route in addition to inflammation-related pathways and that cellular senescence interacts synergistically with the MAPK pathway to further impair the regular synthesis of cholesterol and androgens (54). It is well recognized that androgens and BPH are closely related (55). High estrogen may cause bladder overactivity by activating the RhoA/ROCK pathway, and altered estrogen/androgen ratios are associated with BPH (56). Estrogen has a pro-inflammatory effect on the prostate, and in men, the combined effects of inflammation, dyslipidemia, and a sex steroid environment can have an impact on the start and progression of BPH (57). A study showed worse prognosis and mortality in SARS-CoV-2 infected men with low testosterone levels (58). This implies that sex steroid hormones represent a significant relationship between COVID-19 and BPH and require more investigation. These common DEGs may also contribute to some chronic inflammatory conditions, like inflammatory bowel disease (IBD), and research points to a potential co-regulatory link between IBD and COVID-19 (59). Altered levels of the enzyme angiotensin converting enzyme 2 (ACE2) may be a co-pathogenic factor in COVID-19 and IBD. If immunotherapy is given to patients with IBD, it may increase the chance of SARS-CoV-2 infection (60). Therefore, further studies are needed for patients with both IBD and COVID-19.

The results of GO analysis showed that the immunological response was the main pathway of the BPs of these common DEGs. The symptoms of SARS-CoV-2 infection are exacerbated by the activation of inflammatory responses, particularly interferon responses (61); when interferon expression is ineffective, SARS-CoV-2 replicates widely, triggering an inflammatory response. This is the case in some people with severe COVID-19 who have delayed or no induction of interferon-I and -III (62). CCs of common DEGs that are primarily concentrated in the secretory granule lumen, the vesicle lumen, and the cytoplasmic vesicle lumen, all of which have been linked to immune cell activity. MFs of common DEGs focus on enzyme inhibitor activity. Recent investigations have discovered that the co-expression of ACE2 and TMPRSS2 in an organ is crucial for viral infection of that organ (63). It is vital to further examine whether the virus affects these organs when ACE2 and TMPRSS2 are co-expressed in other organs such as the testes and prostate (64). One of the essential components of the ACE2/Ang-(1-7)/Mas system, ACE2 is closely related to SARS-CoV-2 infection (64). By reducing Ang-II inflammation and proliferation, it has anti-inflammatory actions. Additionally, Ang (1–7) can reduce inflammation by blocking the NF-B pathway and cytokines (65). Inhibition of ACE2 caused by SARS-CoV-2 infection may activate pro-inflammatory pathways and increase cytokine production, resulting in an inflammatory response in the prostate and worsening of BPH.

In addition, we performed a gene-disease analysis in which there were chronic diseases, including hypertension and diabetes. Angiotensin-converting enzyme inhibitors (ACEI) can reduce excessive inflammation and increase intracellular antiviral responses, while in patients with COVID-19, hypertension inhibits viral clearance and worsens excessive airway inflammation (66). High blood sugar levels may raise the risk of mortality from COVID-19 in diabetics (67). There are also prostatic neoplasms, colonic neoplasms, and T-cell lymphoma/leukemia. A previous study (42) showed that COVID-19 is associated with various tumors, including breast cancer, malignant lymphoma, lymphocytic disorders, and leukemia, which is consistent with our findings. Patients with tumors may be more likely to pass away due to their deteriorating health from the SARS-CoV-2 infection.

We screened five hub genes (BIRC5, DNAJC4, DTL, LILRB2, and NDC80) using three machine learning algorithms, and the other four genes had good diagnostic properties in both the training and validation sets, with the exception of BIRC5, which had an AUC of only 0.542 in the validation set GSE132714 of BPH. We speculated that this may be because the sample size of GSE132714 is too small and further studies are still needed in the future. The apoptosis inhibitory protein family member Survivin can be encoded by BIRC5. According to Beding et al. (68), BIRC5 is highly expressed in 16 different malignancies, including prostate cancer (Pca), and may be used as a diagnostic marker for a number of tumor types. High expression of BIRC5 has been associated with a worse prognosis, tumor stage, and response to therapy in survival and clinicopathology studies. In patients with non-small cell lung cancer with COVID-19, BIRC5, a member of the inhibitor of apoptosis (IAP) gene family, may be a target gene with important predictive significance (69). Chronic inflammation, which has been associated with the formation and progression of Pca, is related to both precancerous and malignant Pca. Myeloid cells, macrophages, and lymphocyte recruitment and growth in the prostate gland can promote DNA double-strand breaks and androgen receptor activation in prostate epithelial cells, accelerating tumor development (70). The proteins encoded by DTL participate in several processes, such as translesion production, control of the G2/M transition of the mitotic cell cycle, and protein ubiquitination. Prior research has shown that DTL may function as a biomarker for COVID-19 (71). LILRB2, a Class I MHC antigen receptor, is implicated in the suppression of immunological responses and the development of tolerance. The decrease of LILRB2 in peripheral blood mononuclear cells (PBMC) suggests that LILRB2 may be a new target to overcome immune evasion and improve vaccination strategies (72). NDC80 is necessary for normal chromosomal segregation, a process closely related to mitosis, and serves to organize and regulate microtubule-kinetochore interactions (73). Aneuploidy development, which is linked to tumors, would result from overexpression of NDC80 because it would interfere with microtubule dynamics and chromosomal segregation in mitosis (74). The cell cycle and cell proliferation in Pca are tightly correlated with the NDC80-related gene Spindle pole body component 25 (SPC25) (75, 76). Previous studies have shown that glucocorticoids can act by modulating DNAJC4. In the treatment of COVID-19, a previous study demonstrated that glucocorticoids can accelerate recovery times and lower hospitalizations (77). Therefore, we hypothesize that DNAJC4 may be an important biological target for glucocorticoids in the treatment of COVID-19 and its complications. The identification of these molecular markers can open up new possibilities for the identification and care of BPH patients who have COVID-19 infections.

It is well known that the development of COVID-19 is highly correlated with immune cell performance. Important components of the adaptive immune system that are crucial in preventing the majority of viral infections are B cells, CD4 T cells, and CD8 T cells (78). Substantial drop in total T cell, CD8 or CD4 T cell counts, especially in the sickest COVID-19 patients (79). Chronic inflammation and immunological dysregulation contribute to the progression of BPH, and *in vitro* research has shown that the administration of dihydrotestosterone, which inhibits CD4 T cells’ production of pro-inflammatory cytokines, has an immunomodulatory effect (55). In COVID-19, Tregs may have negative consequences by directly promoting inflammation in the most severe phases of the disease and blocking antiviral T-cell responses (80). A new strategy for the treatment and prevention of BPH in clinical practice may be offered by the use of Tregs as cells to reduce inflammation in BPH through CD39 (81). In severe COVID-19, NK cells have impaired antifibrotic function, which may be connected to the development of fibrotic lung disease. NK cells are active against SARS-CoV-2 but perform poorly when COVID-19 is severe (82). Based on the results of our immune infiltration analysis, five hub genes are closely associated with several of the immune cells above and may play key roles in the pathogenesis of BPH and COVID-19. Five hub genes were differentially expressed in COVID-19 patients compared to controls, and they were associated with the activation of regulatory T cells, NK cells, and CD4 memory T cells. Immune infiltration analyses revealed that these three immune cells were differentially expressed in the COVID-19 group and controls. Therefore, we speculate that the five hub genes may influence these three immune cells, which in turn may change the immunological state and inflammatory response.

MiRNAs (83) can regulate target genes and have a significant impact on a variety of biological processes. TFs are proteins that bind to certain DNA sequences to control transcription and gene expression. By binding to particular gene sequences, TFs can perform a crucial function (84). We identified multiple potential drugs that can influence patients with BPH infected with COVID-19. It has been demonstrated that testosterone reduces symptoms through upregulating anti-inflammatory cytokines, downregulating pro-inflammatory cytokines, and changing immunological function (85). Rat prostate weight and testosterone levels can both be decreased by phytoestrogens, according to an animal experiment (86). Phytoestrogens may also have anti-COVID-19 actions and inhibit the adhesion of SARS-CoV-2 to host cells (87). Etoposide causes prostate hyperplasia cells to undergo apoptosis (88). Etoposide, in the meantime, may be used as a salvage therapy to treat the cytokine storm of COVID-19 (89). Rapamycin is a common antifungal drug that may play a role in benign prostatic hyperplasia in rats by affecting autophagy (90). Numerous studies have shown the significance of rapamycin in the prevention and treatment of COVID-19 because it is a mTOR inhibitor and the mTOR pathway plays a significant role in the development and replication of SARS-CoV-2 (91). Genistein may play a role in BPH by inhibiting α1-adrenergic, non-adrenergic, and neurogenic human prostate smooth muscle contraction and stromal cell growth (92). Additionally, genistein may have significant antiviral effects as a strong protease inhibitor of SARS-CoV-2 (93). The predicted drugs mentioned above in COVID-19 with BPH still require more research.

In conclusion, our study has several advantages. First, we are the first to screen DEGs and investigate common biological functions using the COVID-19 and BPH datasets from open databases. Second, we used three machine learning methods to search for hub genes, and two datasets were used to confirm the diagnosis accuracy of hub genes. Also, we investigated the association between hub genes and immune cells using the CIBERSORT approach. Finally, we also predicted how gene transcription levels would be regulated and potential small-molecule medicines. Despite the fact that our study is convincing, it has several limitations. Our work did not use *in vivo* or *in vitro* validation experiments; instead, it merely used data from public databases to conduct investigations to find prospective biomarkers. Second, more research needs to be done on the molecular mechanism by which COVID-19 is connected to BPH. In the future, we will conduct more studies to demonstrate the potential role of these hub genes in COVID-19 and BPH.

## 5 Conclusion

Bioinformatics research of the COVID-19 and BPH databases revealed the biological relationship between COVID-19 and BPH. This study also provided some information on the pathogenesis of COVID-19 and BPH, which confirms the function of inflammation-related pathways and immune cells. Additionally, this study provides prospective small-molecule drugs for therapeutic use. This is crucial to understand the potential common pathogenic and susceptibility pathways between them.

## Data availability statement

The original contributions presented in the study are included in the article/**Supplementary Material**. Further inquiries can be directed to the corresponding author.

## Ethics statement

Our data on human participants were obtained from the GEO database, accession numbers GSE157103, GSE166253, GSE7307, and GSE132714.

## Author contributions

HZ and MX designed the study and wrote the manuscript; PH and YL performed bioinformatic analysis of the data; CR, ML, and YP collated the data. SW and XL reviewed and revised the final manuscript of the article. All authors contributed to the article and approved the submitted version.
