# Supplementary material for: Identifying hub genes and common biological pathways between COVID-19 and benign prostatic hyperplasia by machine learning algorithms
Source: Front Immunol. 2023 Jun 23;14:1172724. doi: 10.3389/fimmu.2023.1172724 (PMC10328422; doi:10.3389/fimmu.2023.1172724)
Supplement: Supplementary file 1 [file DataSheet_1.docx]

Identifying Hub Genes and Common Biological Pathways Between COVID-19 and Benign Prostatic Hyperplasia by Machine Learning Algorithms

Hang Zhou^1†^, Mingming Xu^1†^, Ping Hu^2†^, Yuezheng Li^1^, Congzhe Ren^1^, Muwei Li^1^, Yang Pan^1^, Shangren Wang^1^, and Xiaoqiang Liu^1*^

^†^Hang Zhou, Mingming Xu, and Ping Hu contributed equally to this work.

^1^Department of Urology, Tianjin Medical University General Hospital, 154 Anshan Road, Heping District, Tianjin, 300052, China

^2^Department of Orthopedics, Tianjin Medical University General Hospital, 154 Anshan Road, Heping District, Tianjin, 300052, China

*Correspondence

Xiaoqiang Liu

Email: xiaoqiangliu1@163.com

Keywords: COVID-19, benign prostatic hyperplasia, functional enrichment, hub genes, machine learning algorithms.


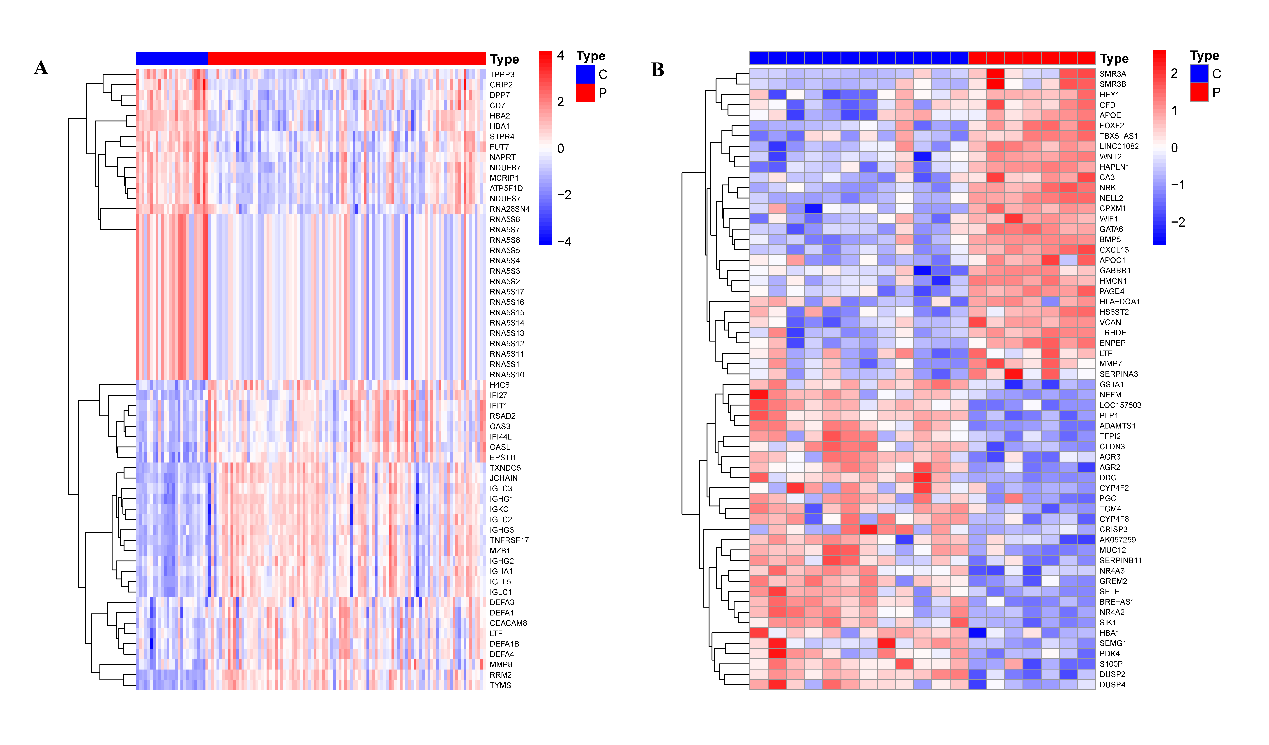


**Figure S1.** The heatmaps show DEGs of (A) COVID-19 (GSE157103) and (B) BPH (GSE7307)


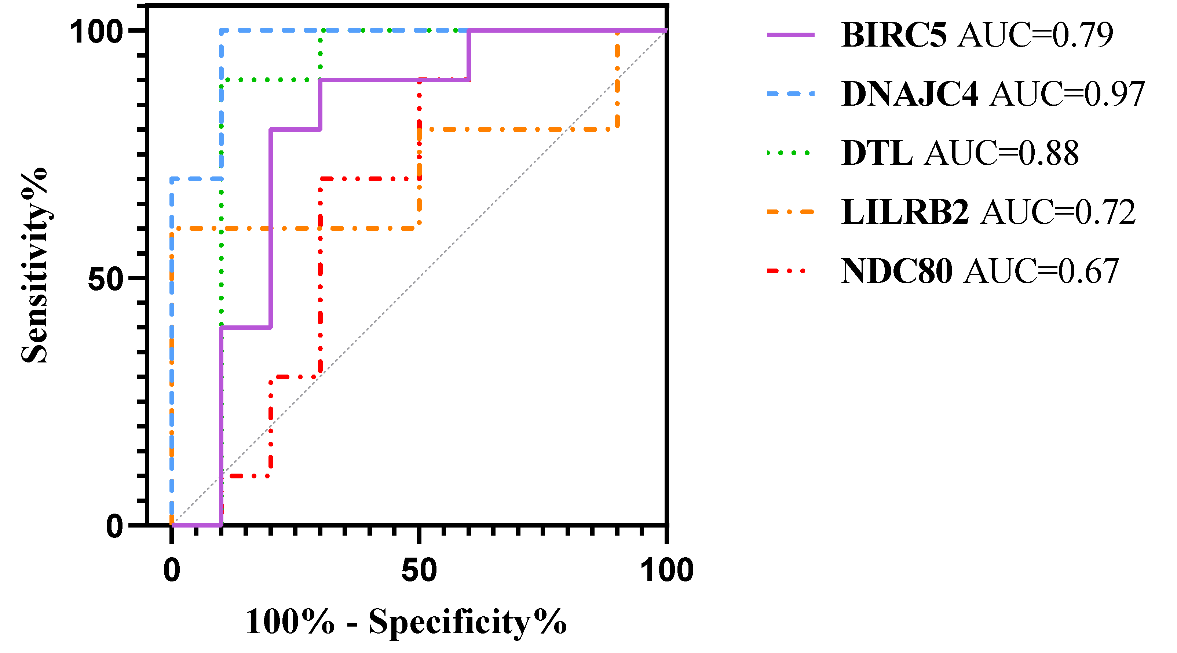


**Figure S2.** ROC curves of five hub genes in the COVID-19 validation dataset (GSE166253).


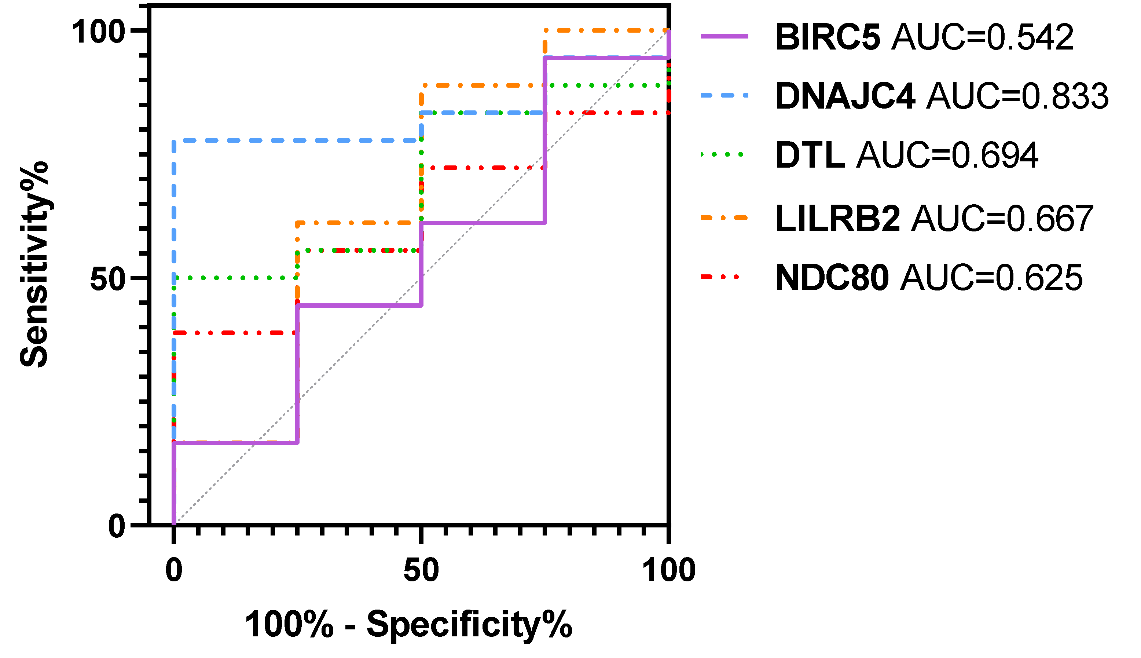


**Figure S3.** ROC curves of five hub genes in the BPH validation dataset (GSE132714).


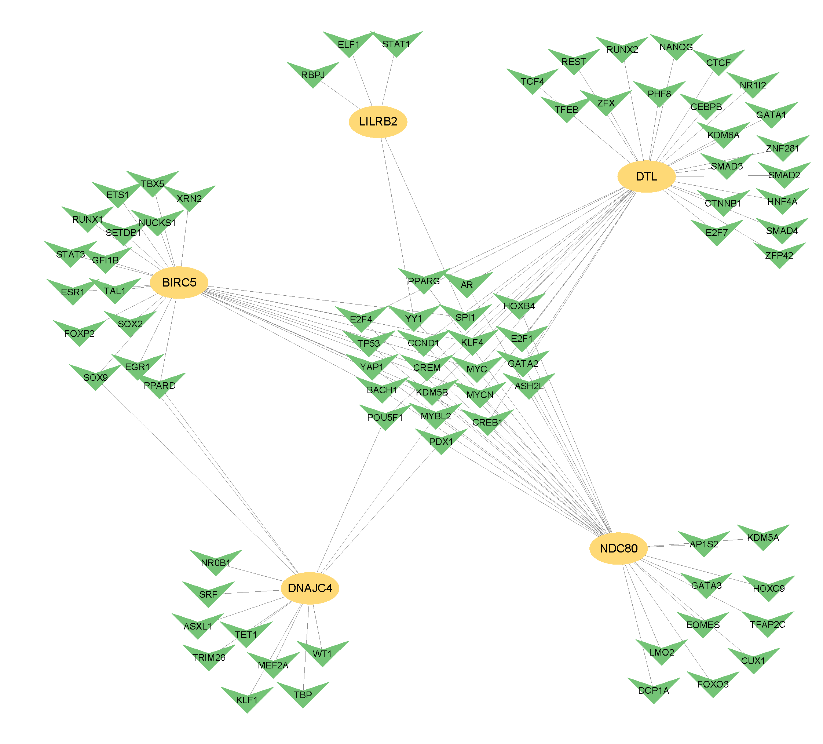


**Figure S4.** Construction of a regulatory interaction network for DEG-TFs.


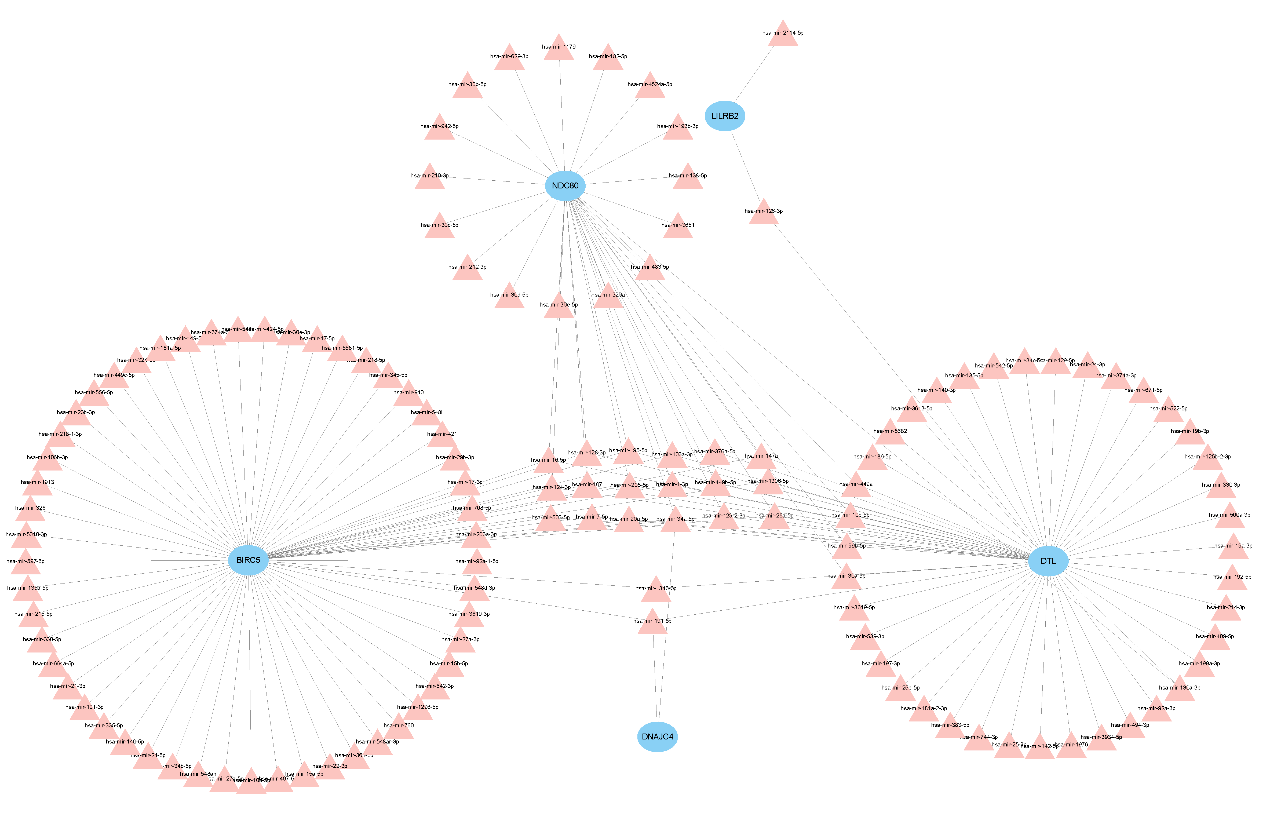


**Figure S5.** The construction of a regulatory interaction network for DEG-miRNAs.
